# Baseline characteristics and outcome of stroke patients after endovascular therapy according to previous symptomatic vascular disease and sex

**DOI:** 10.3389/fneur.2024.1293905

**Published:** 2024-04-17

**Authors:** Marieta Peycheva, Giovanna Padlina, Kotryna Genceviciute, Marina P. Krasteva, Anna Boronylo, Martina B. Goeldlin, Madlaine Müller, Elena S. Wenz, Mandy D. Müller, Helly Hammer, Philipp Bücke, Sandra Bigi, Barbara Goeggel Simonetti, Angelika Hoffmann, Roza M. Umarova, Sara Pilgram-Pastor, Jan Gralla, Pasquale Mordasini, Kateryna Antonenko, Mirjam R. Heldner

**Affiliations:** ^1^Department of Neurology, Inselspital, University Hospital and University of Bern, Bern, Switzerland; ^2^Department of Neurology and Research Institute, Medical University Plovdiv, Plovdiv, Bulgaria; ^3^Clinica Luganese, Mancucco, Lugano, Switzerland; ^4^Department of Neurology, University Hospital Queen Giovanna, Sofia, Bulgaria; ^5^Institute of Diagnostic and Interventional Neuroradiology, Inselspital, University Hospital and University of Bern, Bern, Switzerland; ^6^Department of Neurosurgery, Inselspital, University Hospital and University of Bern, Bern, Switzerland; ^7^Division of Paediatric Neurology, Department of Paediatrics, Children's Hospital Lucerne, Lucerne, Switzerland; ^8^Institute of Social and Preventive Medicine (ISPM), University of Bern, Bern, Switzerland; ^9^Division of Neuropaediatrics, Istituto Pediatrico della Svizzera Italiana IPSI EOC, Ospedale Regionale di Bellinzona e Valli, Bellinzona, Switzerland; ^10^Faculty of Biomedical Sciences, Università della Svizzera Italiana, Lugano, Switzerland; ^11^Netzwerk Radiologie, Kantonsspital St. Gallen, St. Gallen, Switzerland

**Keywords:** cerebrovascular disease/stroke, acute stroke therapy, atherosclerosis, endovascular treatment, large vessel occlusion

## Abstract

**Aim:**

The aim of this study was to investigate baseline characteristics and outcome of patients after endovascular therapy (EVT) for acute large vessel occlusion (LVO) in relation to their history of symptomatic vascular disease and sex.

**Methods:**

Consecutive EVT-eligible patients with LVO in the anterior circulation admitted to our stroke center between 04/2015 and 04/2020 were included in this observational cohort study. All patients were treated according to a standardized acute ischaemic stroke (AIS) protocol. Baseline characteristics and successful reperfusion, recurrent/progressive in-hospital ischaemic stroke, symptomatic in-hospital intracranial hemorrhage, death at discharge and at 3 months, and functional outcome at 3 months were analyzed according to previous symptomatic vascular disease and sex.

**Results:**

995 patients with LVO in the anterior circulation (49.4% women, median age 76 years, median admission NIHSS score 14) were included. Patients with multiple vs. no previous vascular events showed higher mortality at discharge (20% vs. 9.3%, _age/sex − adjusted_OR = 1.43, *p* = 0.030) and less independency at 3 months (28.8% vs. 48.8%, _age/sex − adjusted_OR = 0.72, *p* = 0.020). All patients and men alone with one or multiple vs. patients and men with no previous vascular events showed more recurrent/progressive in-hospital ischaemic strokes (19.9% vs. 6.4% in all patients, _age/sex − adjusted_OR = 1.76, *p* = 0.028) (16.7% vs. 5.8% in men, age-adjustedOR = 2.20, *p* = 0.035). Men vs. women showed more in-hospital symptomatic intracranial hemorrhage among patients with one or multiple vs. no previous vascular events (23.7% vs. 6.6% in men and 15.4% vs. 5.5% in women, OR = 2.32, *p* = 0.035/_age − adjusted_OR = 2.36, *p* = 0.035).

**Conclusions:**

Previous vascular events increased the risk of in-hospital complications and poorer outcome in the analyzed patients with EVT-eligible LVO-AIS. Our findings may support risk assessment in these stroke patients and could contribute to the design of future studies.

## Introduction

Multiple vascular events are predominantly driven by atherosclerosis, especially when cardiac and peripheral locations are affected (1–3).

Patients who suffer an acute ischaemic stroke (AIS) and have a history of multiple vascular events have certain characteristics: they tend to be older, have multiple vascular risk factors and often have more than one possible cause for their stroke (3). Despite the implementation of more aggressive preventive measures, these patients remain more likely to have complications and are at higher risk of recurrent vascular events and vascular-related mortality (3). Notably, previous studies looking at pre-existing vascular disease focused primarily on patients with transient ischaemic attack (TIA) and/or mild to moderate AIS (3–6).

Past studies have uncovered significant sex differences in vascular risk factor profiles. These studies have also identified the impact of specific vascular risk factors such as atrial fibrillation, diabetes mellitus, and arterial hypertension in women (7, 8). Women have been reported to develop AIS at an older age compared to men (7, 8). Furthermore, they are less likely to receive intravenous thrombolysis (IVT) (9), and tend to experience more severe AIS with worse outcomes and higher mortality rates (8, 10, 11). However, data on sex differences in patients eligible for endovascular therapy (EVT) with large vessel occlusion (LVO)-AIS remain limited (12).

The aim of this study was to investigate baseline characteristics and outcome after EVT of patients with LVO-AIS according to history of symptomatic vascular disease and sex.

## Methods

**Figure 1:**
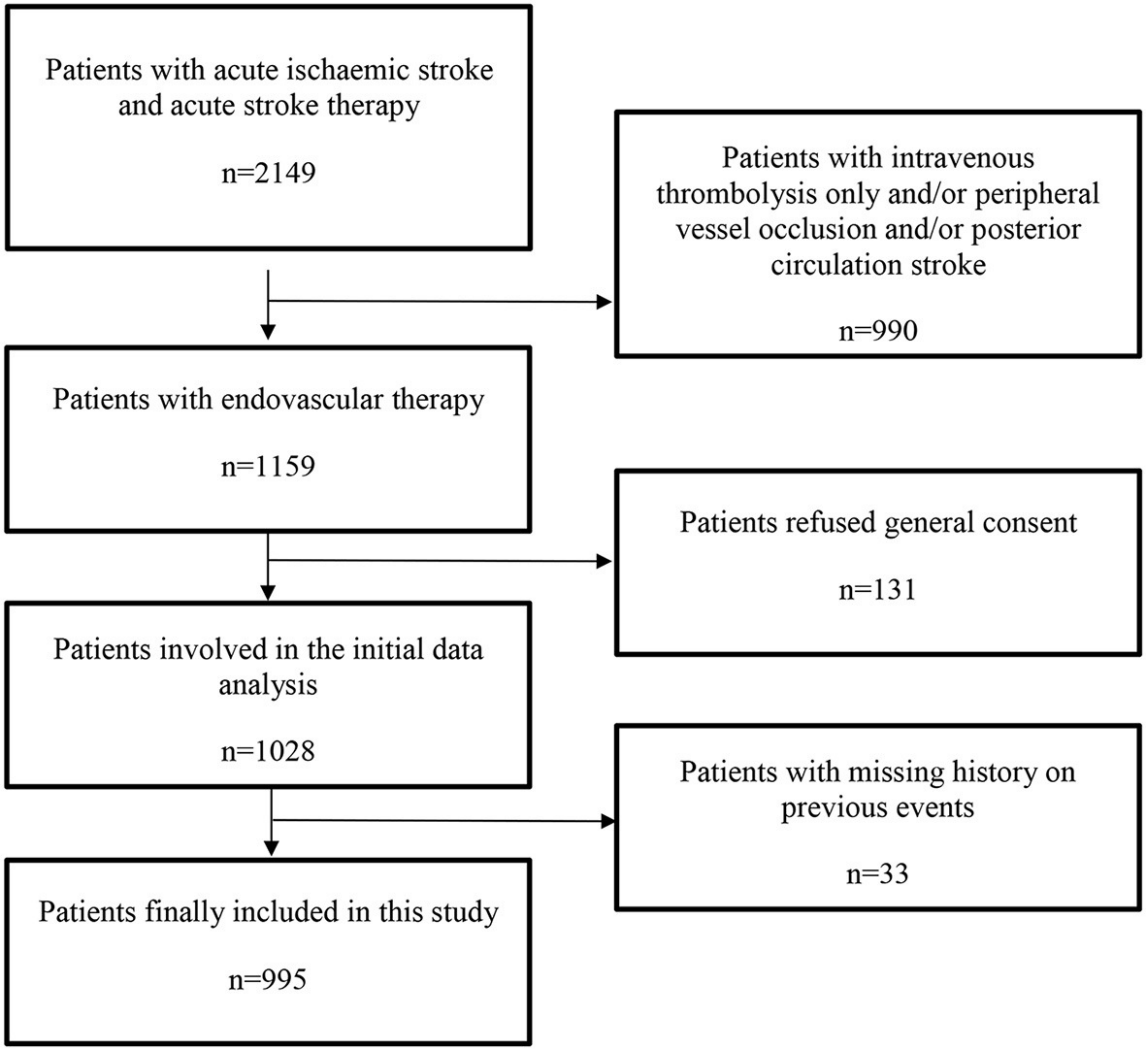
Flow chart of patients who met inclusion and exclusion criteria.

Data were extracted from AIS patients treated between April 2015 and April 2020 from the prospective Bern Stroke Center Registry and retrospectively analyzed. We included all patients with AIS and acute large vessel occlusion (LVO) in the anterior circulation who were treated with EVT. AIS was defined according to the American Stroke Association/American Health Association (ASA/AHA) criteria (13). LVO was defined as acute vessel occlusion of the internal carotid artery (ICA), the carotid terminus (ICA-T), the proximal middle cerebral artery (M1- or M2-segment; MCA) or tandem occlusion (ICA and M1- or M2-segment of the ipsilateral MCA). Patients who were treated with intravenous thrombolysis only and/or had suffered peripheral vascular occlusion and/or posterior circulation stroke were excluded from the study. A flow chart showing the inclusion and exclusion criteria of the study is shown in Figure 1.

Patients were assessed on admission to the emergency department using a standardized AIS protocol that included history taking and clinical examination by a board-certified neurologist, laboratory blood tests, electrocardiography and cranial imaging with CT and/or MR arteriography.

The decision for or against EVT was made individually and in consultation with experienced neurologists and neuroradiologists according to international as well as our institutional guidelines (14–16). EVT was performed as early as possible after diagnosis and after consideration of indications and contraindications. All patients underwent diagnostic digital subtraction angiography. Two independent neuroradiologists evaluated the radiological data. After EVT, all patients were hospitalized in the stroke unit, intermediate or intensive care unit at the Bernese stroke center.

A follow-up CT and/or MR arteriography was performed 24 h after EVT or at an earlier time point if secondary neurological deterioration occurred.

A 3-month follow-up was performed clinically by a board-certified neurologist or by telephone by a trained study nurse.

Patients were a priori classified according to history of symptomatic vascular disease in different vascular beds: peripheral, coronary or cerebrovascular, if leading to past hospitalization. Patients were attributed to previous *coronary* artery event(s) if there was previous myocardial infarction, unstable angina, angina and previous percutaneous coronary intervention or coronary bypass surgery. History was positive for *peripheral* vascular event(s) with previous aortic aneurysm rupture, aortic dissection, acute limb ischaemia, critical limb ischaemia, acute visceral ischaemia, intermittent claudication, and previous angioplasty or stenting and peripheral arterial bypass grafting or amputation. History was positive for *cerebrovascular* event(s) in case of a previous transient ischaemic attack, AIS or symptomatic intracranial hemorrhage.

Reperfusion was assessed using the modified Thrombolysis in Cerebral Infarction Score (mTICI) (17). Successful reperfusion (SR) was defined as mTICI 2b/3. Recurrent/progressive in-hospital ischaemic stroke was defined according to the ASA/AHA criteria and if NIHSS score increased by at least 4 points. In-hospital symptomatic intracranial hemorrhage (sICH) was defined according to the ECASS II criteria (18). Death was classified as vascular if the patient died within 2 weeks of a vascular event. Functional outcome was assessed according to the modified Rankin Scale (mRS). mRS 0–2 was defined as functional independence (FI) and mRS 0–1 as excellent outcome (EO) (19).

### Statistical analysis

Statistical analysis was performed using SPSS 25.0 (SPSS Inc., Chicago, Illinois, USA). In univariable analysis, the χ^2^-test was applied for categorical variables and the ANOVA-test for ordinal and continuous variables to compare baseline characteristics between patients with one or multiple vs. no vascular events and between patients with multiple vs. no vascular events. Sensitivity analyses were performed for patients according to sex and according to cardioembolism only.

A 2-tailed *p* < 0.05 was considered significant.

Binary logistic regression and ordinal or linear regression analysis were performed for outcome analysis where appropriate. Regression analysis was adjusted for age and sex where appropriate.

### Standard protocol approvals, registrations, patient consent and reporting

The Bernese stroke registry was approved by the local ethics committee (KEK Bern 2016–01905) for quality control and research. Informed consent was waived by the ethics committee. Patients were informed about the registry and the potential use of their data for research. In accordance with Swiss law, patients who refused the use of their data for research were excluded from this analysis. This study complies with the Declaration of Helsinki. Data analyses followed strengthening the reporting of observational studies in epidemiology (STROBE) reporting guidelines.

## Results

### Baseline characteristics of patients according to previous vascular events

A total of 995 patients (49.4% women, median age 76 years, median admission NIHSS score 14) were included in the current study. A total of 610 (61.3%) patients had no previous vascular event, 310 (31.2%) one and 75 (7.5%) multiple (Table 1). Patients with one or multiple previous vascular events were older (median age 80 and 79 years respectively vs. 72.7 in patients with no events, *p* < 0.0001) and less frequently female (41.9% and 37.3% respectively vs. 54.8% in patients with no events, *p* < 0.0001). They more frequently had a history of vascular risk factors, were more likely to be treated with antithrombotics (70.3% and 77.3% vs. 27.4% in patients with no events, *p* < 0.0001) and other (antidiabetic, lipid-lowering and antihypertensive) drugs (*p* < 0.0001) pre-stroke. They more often had higher admission HbA1c values (5.8% and 5.9% vs. 5.7% in patients with no events, *p* < 0.0001), lower LDL values (2.15 mmol/l and 2.1 mmol/l vs. 2.65 mmol/l in patients with no events, *p* < 0.0001) and a different pattern of stroke etiology (*p* < 0.0001) as well as a different applied therapy strategy during emergency care (*p* = 0.043). The three groups were similar with respect to other baseline characteristics such as admission NIHSS, location of main acute vessel occlusion, extra/intracranial therapy modality and EVT duration. All baseline data are shown in Table 1.

**Table 1:** Baseline characteristics of patients overall.

**Baseline characteristics**	**No previous vascular event (*n =* 610)**	**One previous vascular event (*n =* 310)**	**Multiple previous vascular events (*n =* 75)**	***P*-value**
Age (years)	72.7 (19–101)	80 (34–98)	77 (54–93)	< 0.0001
Female	334 (54.8%)	130 (41.9%)	492 (37.3%)	< 0.0001
**Vascular risk factors**				
Arterial hypertension	404 (66.2%)	265 (85.5%)	70 (93.3%)	< 0.0001
Actively smoking or stopped < 2years	135 (22.1%)	51 (16.5%)	18 (24%)	0.096
Diabetes mellitus	99 (16.2%)	90 (29%)	28 (37.3%)	< 0.0001
Dyslipidaemia	381 (62.5%)	239 (77.1%)	70 (93.3%)	< 0.0001
Coronary heart disease	0	185 (59.7%)	67 (89.3%)	< 0.0001
Peripheral artery disease	0	30 (9.7%)	43 (57.3%)	< 0.0001
Atrial fibrillation or flutter	220 (36.1%)	142 (45.8%)	41 (54.7%)	0.001
Previous ischaemic stroke	0	88 (28.4%)	45 (60%)	< 0.0001
Previous haemorrhagic stroke	0	8 (2.6%)	5 (6.7%)	< 0.0001
**Stroke etiology**				< 0.0001
Cardiac embolism	254 (41.6%)	153 (49.4%)	47 (62.7%)	
Cervical artery dissection	27 (4.4%)	1 (0.3%)	0	
Large artery atherosclerosis	85 (13.9%)	35 (11.3%)	8 (10.7%)	
More than one possible etiology	35 (5.7%)	35 (11.3%)	2 (2.7%)	
Other determined etiology	40 (6.6%)	13 (4.2%)	0	
Unknown, complete evaluation	80 (13.1%)	29 (9.4%)	9 (12%)	
Unknown, incomplete evaluation	89 (14.6%)	44 (14.2%)	9 (12%)	
Independency before stroke (mRS 0–2)	516 (84.9%)	247 (79.9%)	59 (78.7%)	0.104
**Pre-stroke antithrombotics**				< 0.0001
Antiplatelets	96 (15.7)	160 (51.6%)	40 (53.3%)	
NOAC	32 (5.2%)	25 (8.1%)	5 (6.7%)	
OAC	36 (5.9%)	25 (8.1%)	8 (10.7%)	
NOAC or OAC and antiplatelets	32 (5.2%)	8 (2.6%)	5 (6.7%)	
None	443 (72.6%)	92 (29.7%)	17 (22.7%)	
**Other pre-stroke drugs**				
Antidiabetic treatment	52 (8.5%)	52 (16.8%)	16 (21.3%)	< 0.0001
Lipid-lowering drugs	80 (13.1%)	141 (45.5%)	49 (65.3%)	< 0.0001
Antihypertensives	333 (54.7%)	239 (77.1%)	66 (88%)	< 0.0001
Admission systolic blood pressure (mmHg)	156 (60–265)	156 (80–253)	159 (101–239)	0.848
Admission NIHSS score	14 (0–36)	14 (0–36)	15 (1–36)	0.619
**Admission laboratory values**				
HbA1c (%)	5.7 (4.1–12.8)	5.8 (4.7–12.9)	5.9 (4.2–10.1)	< 0.0001
LDL (mmol/l)	2.65 (0.5–8)	2.15 (0.5–6.4)	2.1 (0.3–5.1)	< 0.0001
CRP (mmol/l)	3 (0–380)	4 (1–145)	5 (3–198)	0.435
Wake-up stroke	163 (26.7%)	78 (25.2%)	17 (22.7%)	0.701
Known onset to groin puncture time (min.)	200 (74–1436)	193 (80–1283)	183 (70–614)	0.178
**Location of main acute vessel occlusion**				0.526
ICA	71 (11.6%)	30 (9.7%)	12 (16%)	
Carotid-T	49 (8%)	22 (7.1%)	6 (8%)	
ICA and M1/2-segment of MCA	51 (8.4%)	23 (7.4%)	4 (5.3%)	
M1-segment of MCA	290 (47.5%)	147 (47.4%)	40 (53.3%)	
M2-segment of MCA	149 (24.4%)	88 (28.4%)	13 (17.3%)	
**Therapy modality**				0.043
MT only	301 (49.3%)	154 (49.7%)	42 (56%)	
MT and IVT	283 (46.4%)	152 (49%)	28 (37.3%)	
MT and IAT	26 (4.3%)	4 (1.3%)	5 (6.7%)	
Stent retriever applied	575 (94.3%)	287 (92.6%)	67 (89.3%)	0.216
**Therapy modality**				
Intracranial	19 (3.1%)	4 (1.3%)	3 (4%)	0.192
Extracranial	101 (16.6%)	33 (10.6%)	10 (13.3%)	0.053
EVT duration	55 (9–412)	52 (10–237)	45 (20–186)	0.086

For categorical variables, the number of patients and percentage in brackets are shown. For non-normally distributed continuous and ordinal variables, median, minimum and maximum ranges are shown (in brackets). For normally distributed continuous and ordinal variables, average and standard deviation are shown (in brackets). NOAC, new oral anticoagulants; OAC, oral anticoagulants; ICA, internal carotid artery; MCA, middle cerebral artery; EVT, endovascular therapy; MT, mechanical thrombectomy; IVT, intravenous thrombolysis with rtPA; IAT, intraarterial thrombolysis with urokinase.

### Outcome characteristics of patients according to previous vascular events

**Figure 2:**
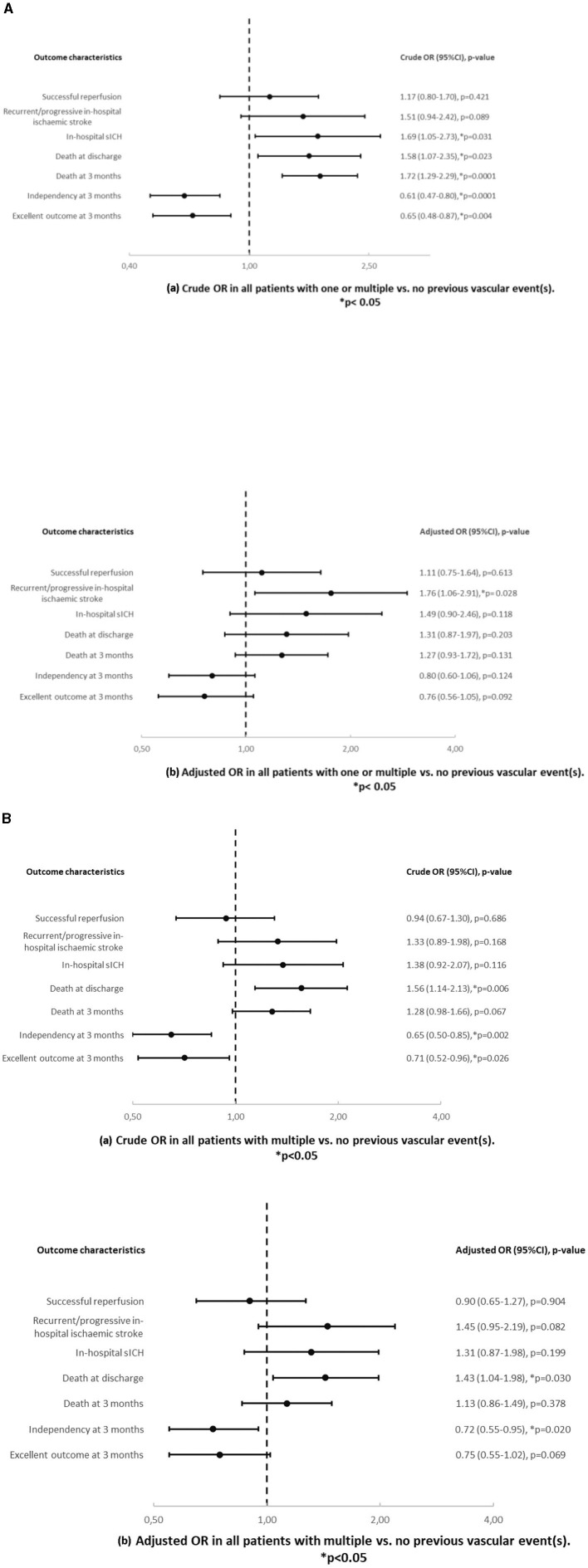
**(A)** Crude (a) and adjusted (b) odds ratios and *p*-values in all patients with one or multiple vs. no previous vascular event(s). Results are adjusted for age and sex. **(B)** Crude (a) and adjusted (b) odds ratios and *p*-values in all patients with multiple vs. no previous vascular event(s). Results are adjusted for age and sex.

In univariable analysis (Table 2, Figure 2A), patients with one or multiple vs. no previous vascular events showed more in hospital sICH (20.2% vs. 6%, OR = 1.69, *p* = 0.031), death at discharge (22.6% vs. 9.3%, OR = 1.58, *p* = 0.023) and at 3 months (69.3% vs. 22.7%, OR = 1.72, *p* < 0.0001), less independency (67.8% vs. 48.8%, OR = 0.61, *p* < 0.0001) and less excellent outcome at 3 months (43.7% vs. 32.1%, OR = 0.65, *p* = 0.004) and a worse mRS shift at 3 months (*p* < 0.0001). Patients with multiple vs. no previous vascular events (Table 2, Figure 2B) showed higher mortality at discharge (20% vs 9.3%, OR = 1.56, *p* = 0.006) and less independency (28.8% vs. 48.8%, OR = 0.65, *p* = 0.002) and less excellent outcome at 3 months (19.2% vs. 32.1%, OR = 0.71, *p* = 0.026) and a worse mRS shift at 3 months (*p* = 0.005).

**Table 2:** Outcome characteristics of patients overall.

**Outcome characteristics**	**No previous vascular event**	**One previous vascular event**	**Multiple previous vascular events**
Successful reperfusion	523 (85.7%)	274 (88.4%)	63 (84%)
Recurrent/progressive in-hospital ischaemic stroke	39 (6.4%)	28 (9.1%)	8 (10.8%)
In-hospital sICH	36 (6%)	29 (9.4%)	8 (10.8%)
Duration acute care (days)	4 (0–59)	3 (1–35)	4 (0–23)
Death at discharge	57 (9.3%)	39 (12.6%)	15 (20%)
Death at 3 months	136 (22.7%)	102 (33.9%)	24 (32.4%)
**mRS at 3 months**	3 (0–6)	3 (0–6)	4 (0–6)
0	82 (14%)	33 (11.4%)	6 (8.2%)
1	106 (18.1%)	38 (13.1%)	8 (11%)
2	98 (16.7%)	41 (14.1%)	7 (9.6%)
3	79 (13.5%)	37 (12.8%)	14 (19.2%)
4	68 (11.6%)	26 (9%)	10 (13.7%)
5	17 (2.9%)	13 (4.5%)	4 (5.5%)
6	136 (23.2%)	102 (35.2%)	24 (32.9%)
**Death causes**			
Vascular	76 (55.9%)	60 (58.8%)	15 (62.5%)
Non-vascular	16 (11.8%)	9 (8.8%)	3 (12.5%)
Unknown	44 (32.4%)	33 (32.4%)	6 (25%)
Independency at 3 months	286 (48.8%)	113 (39%)	21 (28.8%)
Excellent outcome at 3 months	188 (32.1%)	71 (24.5%)	14 (19.2%)

For categorical variables, the number of patients and percentage in brackets are shown. For non-normally distributed continuous and ordinal variables, median, minimum and maximum ranges are shown (in brackets). For normally distributed continuous and ordinal variables, average and standard deviation are shown (in brackets). Results are adjusted for age and sex. sICH, symptomatic intracerebral hemorrhage; mRS, modified Rankin scale.

In multivariable analysis (Table 2, Figures 2A, B), patients with one or multiple vs. no previous vascular events showed more recurrent/progressive in-hospital ischaemic strokes (19.9% vs. 6.4%, _age/sex − adjusted_OR = 1.76, *p* = 0.028) but otherwise similar outcomes. Patients with multiple vs. no previous vascular events showed higher mortality at discharge (20% vs. 9.3%, _age/sex − adjusted_ OR = 1.43, *p* = 0.030) and less independency at 3 months (28.8% vs. 48.8%, _age/sex − adjusted_OR = 0.72, *p* = 0.020) but otherwise similar outcomes.

### Baseline characteristics of patients according to sex

Women with one or multiple previous vascular events were older (median age 82 and 82 years respectively vs. 76 years in patients with no events, *p* < 0.001), more frequently had a history of vascular risk factors, of pre-stroke antithrombotics (69.2% and 64.3% respectively vs. 28.4% in patients with no events, *p* < 0.0001) and other drugs (p ≤ 0.013), of higher admission HbA1c values (5.8% and 6.1% vs. 5.7% in patients with no events, *p* = 0.014) and lower LDL values (2.3 mmol/l and 2.3 mmol/l vs. 2.7 mmol/l, *p* = 0.001). All other baseline characteristics did not differ between women in this group comparison (Table 3).

**Table 3:** Baseline characteristics of women vs. men.

	**Women**				**Men**			
**Baseline characteristics**	**No previous vascular event (*****n** =* **334)**	**One previous vascular event (*****n** =* **130)**	**Multiple previous vascular events (*****n** =* **28)**	***P*** **-value**	**No previous vascular event (*****n** =* **610)**	**One previous vascular event (*****n** =* **310)**	**Multiple previous vascular events (*****n** =* **75)**	***P*** **-value**
Age (years)	76 (26–101)	82 (34–98)	82 (59–93)	< 0.0001	67 (19–96)	78 (46–94)	76 (54–92)	0.079
**Vascular risk factors**								
Arterial hypertension	236 (70.7%)	109 (83.8%)	26 (92.9%)	0.001	168 (60.9%)	156 (86.7%)	44 (93.6%)	< 0.0001
Actively smoking or stopped < 2 years	52 (15.6%)	19 (14.6%)	5 (17.9%)	0.906	83 (30.1%)	32 (17.8%)	13 (27.7%)	0.012
Diabetes mellitus	52 (15.6%)	40 (30.8%)	11 (39.3%)	< 0.0001	47 (17%)	50 (27.8%)	7 (36.2%)	0.002
Dyslipidaemia	218 (65.3%)	96 (73.8%)	25 (89.3%)	0.001	163 (59.1%)	143 (79.4%)	45 (95.7%)	< 0.0001
Coronary heart disease	0	63 (48.5%)	22 (78.6%)	< 0.0001	0	122 (67.8%)	45 (95.7%)	< 0.0001
Peripheral artery disease	0	14 (10.8%)	22 (78.6%)	< 0.0001	0	16 (8.9%)	21 (44.7%)	< 0.0001
Atrial fibrillation or flutter	133 (39.8%)	71 (54.6%)	20 (71.4%)	< 0.0001	87 (31.5%)	71 (39.4%)	21 (44.7%)	0.098
Previous ischaemic stroke	0	50 (38.5%)	16 (57.1%)	< 0.0001	0	38 (21.1%)	29 (61.7%)	< 0.0001
Previous haemorrhagic stroke	0	3 (2.3%)	1 (3.6%)	0.011	0	5 (2.8%)	4 (8.5%)	< 0.0001
**Stroke etiology**				0.168				< 0.0001
Cardiac embolism	151 (45.2%)	73 (56.2%)	20 (71.4%)		103 (37.3%)	80 (44.4%)	27 (57.4%)	
Cervical artery dissection	6 (1.8%)	0	0		21 (7.6%)	1 (0.6%)	0	
Large artery atherosclerosis	34 (10.2%)	11 (8.5%)	3 (10.7%)		51 (18.5%)	24 (13.3%)	5 (10.6%)	
More than one possible etiology	24 (7.2%)	13 (10%)	1 (3.6%)		11 (4%)	22 (12.2%)	1 (2.1%)	
Other determined etiology	26 (7.8%)	6 (4.6%)	0		14 (5.1%)	7 (3.9%)	0	
Unknown, complete evaluation	47 (14.1%)	14 (10.8%)	2 (7.1%)		33 (12%)	15 (8.3%)	7 (14.9%)	
Unknown, incomplete evaluation	46 (13.8%)	13 (10%)	2 (7.1%)		43 (15.6%)	31 (17.2%)	7 (14.9%)	
Independency before stroke (mRS 0–2)	272 (81.7%)	96 (73.8%)	21 (75%)	0.149	244 (88.7%)	151 (84.4%)	38 (80.9%)	0.208
**Pre-stroke antithrombotics**				< 0.0001				< 0.0001
Antiplatelets	56 (16.8%)	40 (30.8%)	10 (35.7%)		40 (14.5%)	99 (55%)	30 (63.8%)	
NOAC	19 (5.7%)	12 (9.2%)	1 (3.6%)		13 (4.7%)	13 (7.2%)	4 (8.5%)	
OAC	19 (5.7%)	14 (10.8%)	5 (17.9%)		17 (6.2%)	11 (6.1%)	3 (6.4%)	
NOAC or OAC and antiplatelets	1 (0.3%)	12 (9.2%)	2 (7.1%)		2 (0.7%)	5 (2.8%)	3 (6.4%)	
None	239 (71.6%)	40 (30.8%)	10 (35.7%)		204 (73.9%)	52 (28.9%)	7 (14.9%)	
**Other pre-stroke drugs**								
Antidiabetic treatment	26 (7.8%)	22 (16.9%)	4 (14.3%)	0.013	26 (9.4%)	30 (16.7%)	12 (25.5%)	0.004
Lipid-lowering drugs	53 (15.9%)	51 (39.2%)	16 (57.1%)	< 0.0001	27 (9.8%)	90 (50%)	33 (70.2%)	< 0.0001
Antihypertensives	197 (59.2%)	104 (80%)	23 (82.1%)	< 0.0001	136 (49.3%)	135 (75%)	43 (91.5%)	< 0.0001
Admission systolic blood pressure (mmHg)	157 (60–265)	156 (80–230)	166 (101–210)	0.817	154 (88–252)	156 (90–253)	158 (102–239)	0.974
Admission NIHSS score	14 (0–36)	14 (0–36)	16 (1–36)	0.247	13 (0–36)	14 (0–36)	15 (4–24)	0.716
**Admission laboratory values**								
HbA1c (%)	5.7 (4.4–12.8)	5.8 (4.9–12.9)	6.1 (4.2–9.1)	0.014	5.7 (4.1–12)	5.8 (4.7–10.2)	5.8 (4.5–10.1)	0.005
LDL (mmol/l)	2.7 (0.6–8)	2.3 (0.5–6.4)	2.3 (0.9–5.1)	0.001	2.6 (0.5–5.6)	2.1 (0.5–5.5)	2.0 (0.3–4.5)	< 0.0001
CRP (mmol/l)	4 (2–380)	5 (1–80)	6 (3–198)	0.407	3 (0–208)	3 (1–145)	3 (3–157)	0.957
Wake-up stroke	84 (25.1%)	25 (19.2%)	9 (32.1%)	0.237	79 (28.6%)	53 (29.4%)	8 (17%)	0.217
Known onset to groin puncture time (min.)	205 (77–1309)	185 (87–1283)	160 (70–600)	0.378	195 (74–1436)	205 (80–1247)	183 (105–614)	0.358
**Location of main acute vessel occlusion**				0.114				0.753
ICA	40 (12%)	12 (9.2%)	8 (28.6%)		31 (11.2%)	18 (10%)	4 (8.5%)	
Carotid-T	35 (10.5%)	9 (6.9%)	3 (10.7%)		14 (5.1%)	13 (7.2%)	3 (6.4%)	
ICA and M1/2-segment of MCA	22 (6.6%)	5 (3.8%)	1 (3.6%)		29 (10.5%)	18 (10%)	3 (6.4%)	
M1-segment of MCA	169 (50.6%)	71 (54.6%)	13 (46.4%)		121 (43.8%)	76 (42.2%)	27 (57.4%)	
M2-segment of MCA	68 (20.4%)	33 (25.4%)	3 (10.7%)		81 (29.3%)	55 (30.6%)	10 (21.3%)	
**Therapy modality**				0.673				0.077
MT only	157 (47%)	60 (46.2%)	15 (53.6%)		144 (52.2%)	94 (52.2%)	27 (57.4%)	
MT and IVT	164 (49.1%)	68 (52.3%)	12 (42.9%)		119 (43.1%)	84 (46.7%)	16 (34%)	
MT and IAT	13 (3.9%)	2 (1.5%)	1 (3.6%)		13 (4.7%)	2 (1.1%)	4 (8.5%)	
Stent retriever applied	320 (95.8%)	121 (93.1%)	24 (85.7%)	0.056	255 (92.4%)	166 (92.2%)	43 (91.5%)	0.977
**Therapy modality**								
Intracranial	5 (1.5%)	1 (0.8%)	0	0.678	14 (5.1%)	3 (1.7%)	3 (6.4%)	0.129
Extracranial	30 (9%)	7 (5.4%)	4 (14.3%)	0.227	71 (25.7%)	26 (14.4%)	6 (12.8%)	0.005
EVT duration	49 (13–247)	52 (15–196)	41 (21–117)	0.372	64 (9–412)	54 (10–237)	54 (10–237)	0.012

For categorical variables, the number of patients and percentage in brackets are shown. For non-normally distributed continuous and ordinal variables, median, minimum and maximum ranges are shown (in brackets). For normally distributed continuous and ordinal variables, average and standard deviation are shown (in brackets). NOAC, new oral anticoagulants; OAC, oral anticoagulants; ICA, internal carotid artery; MCA, middle cerebral artery; EVT, endovascular therapy; MT, mechanical thrombectomy; IVT, intravenous thrombolysis with rtPA; IAT, intraarterial thrombolysis with urokinase.

Men with one or multiple previous vascular events, more frequently had a history of vascular risk factors, of pre-stroke antithrombotics (71.1% and 85.1% respectively vs. 26.1% in patients with no events, *p* < 0.0001) and other drugs (*p* ≤ 0.004), they showed higher admission HbA1c values (5.8% and 5.8% respectively vs. 5.7% in patients with no events, *p* = 0.005) and lower LDL values (2.1 mmol/l and 2.2mmol/l respectively vs. 2.6 mmol/l in patients with no events, *p* < 0.0001). They presented a different pattern of stroke etiology (*p* < 0.0001), more permanent extracranial stent placements (*p* = 0.005) and a shorter EVT duration (54 vs. 64 min in patients with no events, *p* = 0.012). All other baseline characteristics did not differ between men in this group comparison (Table 3).

### Outcome characteristics of patients according to sex

**Figure 3:**
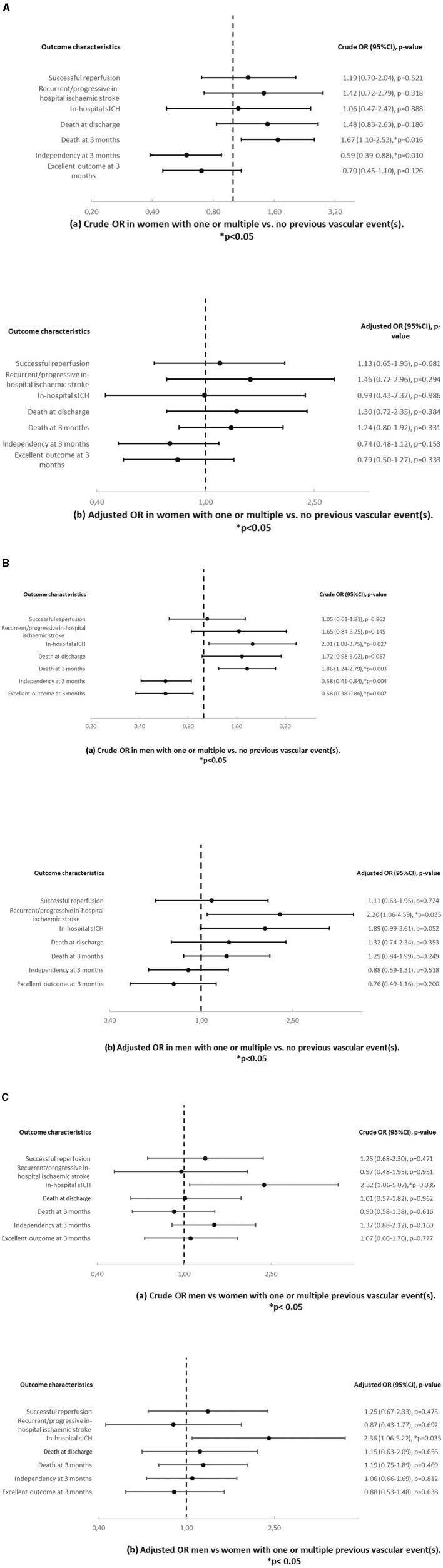
**(A)** Crude (a) and adjusted (b) odds ratios and *p*-values in women with one or multiple vs. no previous vascular event(s). Results are adjusted for age. **(B)** Crude (a) and adjusted (b) odds ratios and p-values in men with one or multiple vs. no previous vascular event(s). Results are adjusted for age. **(C)** Crude (a) and adjusted (b) odds ratios and *p*-values in men vs. women with one or multiple previous vascular event(s). Results are adjusted for age.

In univariable analysis (Table 4 and Figure 3A), women with one or multiple vs. no previous vascular events showed higher mortality at 3 months (73.4% vs. 24.5%, OR = 1.67, *p* = 0.016), less independency at 3 months (59.4% vs. 45.2%, OR = 0.59, *p* = 0.010) and a worse mRS shift at 3 months (OR = 0.007). However, in multivariable analysis, there was no difference of outcome in this group comparison.

**Table 4:** Outcome characteristics of women vs. men.

	**Women**			**Men**		
**Outcome characteristics**	**No previous vascular event**	**One previous vascular event**	**Multiple previous vascular events**	**No previous vascular event**	**One previous vascular event**	**Multiple previous vascular events**
Successful reperfusion	280 (83.8%)	111 (85.4%)	25 (89.3%)	243 (88%)	163 (90.6%)	38 (80.9%)
Recurrent/progressive in-hospital ischaemic stroke	23 (6.9%)	12 (9.3%)	3 (10.7%)	16 (5.8%)	16 (5.8%)	5 (10.9%)
In-hospital sICH	18 (5.5%)	6 (4.7%)	3 (10.7%)	18 (6.6%)	23 (12.8%)	5 (10.9%)
Duration acute care (days)	4 (0–34)	3 (1–32)	4 (1–23)	4 (1–59)	3 (1–35)	4 (0–15)
Death at discharge	33 (9.9%)	15 (11.5%)	7 (25%)	24 (8.7%)	24 (13.3%)	8 (17%)
Death at 3 months	80 (24.5%)	43 (34.1%)	11 (39.3%)	56 (20.7%)	59 (33.7%)	13 (28.3%)
**mRS at 3 months**	3 (0–6)	4 (0–6)	4 (0–6)	2 (0–6)	3 (0–6)	4 (0–6)
0	34 (10.5%)	15 (12.3%)	4 (14.3%)	48 (18.3%)	18 (10.7%)	2 (4.4%)
1	61 (18.9%)	14 (11.5%)	1 (3.6%)	45 (17.1%)	24 (14.3%)	7 (15.6%)
2	51 (15.8%)	12 (9.8%)	2 (7.1%)	47 (17.9%)	29 (17.3%)	5 (11.1%)
3	52 (16.1%)	17 (13.9%)	7 (25%)	27 (10.3%)	20 (11.9%)	7 (15.6%)
4	35 (10.8%)	13 (10.7%)	2 (7.1%)	33 (12.5%)	13 (7.7%)	8 (17.8%)
5	10 (3.1%)	8 (6.6%)	1 (3.6%)	7 (2.7%)	5 (3%)	3 (6.7%)
6	80 (24.8%)	43 (35.2%)	11 (39.3%)	56 (21.3%)	59 (35.1%)	13 (28.9%)
**Death causes**						
Vascular	43 (53.8%)	23 (53.5%)	6 (54.5%)	33 (58.9%)	37 (62.7%)	9 (69.2%)
Non-vascular	8 (10%)	3 (7%)	2 (18.2%)	8 (14.3%)	6 (10.2%)	1 (7.7%)
Unknown	29 (36.3%)	17 (39.5%)	3 (27.3%)	15 (26.8%)	16 (27.1%)	3 (23.1%)
Independency at 3 months	146 (45.2%)	42 (34.4%)	7 (25%)	140 (53.2%)	71 (42.3%)	14 (31.1%)
Excellent outcome at 3 months	95 (29.4%)	29 (23.8%)	5 (17.9%)	93 (35.4%)	42 (25%)	9 (20%)

For categorical variables, the number of patients and percentage in brackets are shown. For non-normally distributed continuous and ordinal variables, median, minimum and maximum ranges are shown (in brackets). For normally distributed continuous and ordinal variables, average and standard deviation are shown (in brackets). sICH, symptomatic intracerebral hemorrhage; mRS, modified Rankin scale.

In univariable analysis (Table 4 and Figure 3B), men with one or multiple vs. no previous vascular events showed more in hospital sICH (23.7% vs. 6.6%, OR = 2.01, *p* = 0.027), death at 3 months (62.0% vs. 20.7%, OR = 1.86, *p* = 0.003), less independency (73.4% vs. 53.2%, OR = 0.58, *p* = 0.004) and excellent outcome (45% vs. 35.4%, OR = 0.58, *p* = 0.007) at 3 months and a worse mRS shift (*p* < 0.0001) at 3 months. In multivariable analysis, men with one or multiple vs. no previous vascular events showed more recurrent/progressive in-hospital ischaemic strokes (16.7% vs. 5.8%, _age − adjusted_OR = 2.20, *p* = 0.035).

In univariable and multivariable analysis (Table 4 and Figure 3C), men vs. women showed more in-hospital sICH among patients with one or multiple vs. no previous vascular events (23.7% vs. 6.6% in men and 15.4% vs. 5.5% in women, OR = 2.32, *p* = 0.035/_age − adjusted_OR = 2.36, *p* = 0.035).

### Outcome characteristics of patients according to cardioembolism only

**Figure 4:**
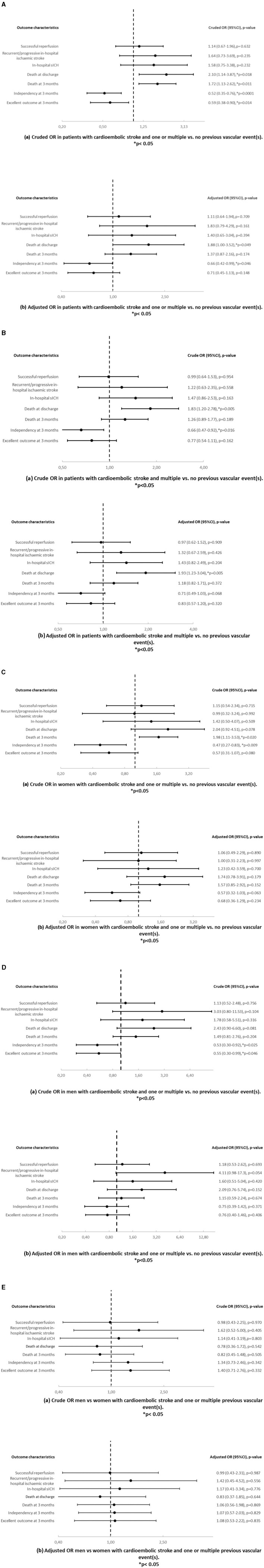
**(A)** Crude (a) and adjusted (b) odds ratios and *p*-values in patients with cardioembolic stroke and one or multiple vs. no previous vascular event(s). Results are adjusted for age and sex. **(B)** Crude (a) and adjusted (b) odds ratios and *p*-values in patients with cardioembolic stroke and multiple vs. no previous vascular event(s). Results are adjusted for age and sex. **(C)** Crude (a) and adjusted (b) odds ratios and *p*-values in women with cardioembolic stroke and one or multiple vs. no previous vascular event(s). Results are adjusted for age. **(D)** Crude (a) and adjusted (b) odds ratios and *p*-values in men with cardioembolic stroke and one or multiple vs. no previous vascular event(s). Results are adjusted for age. **(E)** Crude (a) and adjusted (b) odds ratios and *p*-values in men vs. women with cardioembolic stroke and one or multiple previous vascular event(s). Results are adjusted for age.

Patients with cardioembolic stroke and one or multiple vs. no previous vascular event(s) showed more death at discharge (OR 2.10, *p* = 0.018) and at 3 months (OR 1.72, *p* = 0.011) and less independency (OR 0.52, *p* = 0.0001) and excellent outcome at 3 months (OR 0.59, *p* = 0.014) in univariable analysis (Figure 4A). In multivariable analysis, these patients showed more death at discharge (OR 1.88, *p* = 0.049) and less independency (OR 0.66, *p* = 0.046) at 3 months (OR 0.66, *p* = 0.046). Patients with cardioembolic stroke and multiple vs. no previous vascular event(s) (Figure 4B) had less independency at 3 months (OR 0.66, *p* = 0.016) in univariable analysis. We also observed that these patients experienced more death at discharge in univariable (OR 1.83, *p* = 0.005) and in multivariable analysis (OR 1.93, *p* = 0.005). Women with cardioembolic stroke and one or multiple vs. no previous vascular event(s) showed more death at 3 months (OR 1.98, *p* = 0.020) and less independency (OR 0.47, *p* = 0.009) in univariable analysis (Figure 4C). Men with cardioembolic stroke and one or multiple vs. no previous vascular event(s) also had less independency (OR 0.53, *p* = 0.025) and less excellent outcome (OR 0.55, *p* = 0.046) at 3 months in univariable analysis (Figure 4D). All other analyses were similar (Figures 4A–E).

## Discussion

In this monocentric, retrospectively analyzed, observational cohort study, we investigated baseline characteristics and outcome after EVT of patients with LVO-AIS according to history of symptomatic vascular disease and sex.

The main findings of our study are as follows:

Two out of every five patients in our study had one or multiple previous vascular events. Compared to patients without previous vascular events, these patients had a higher burden of vascular risk factors and use of preventive medications prior to stroke, also when women and men were analyzed separately. In the overall study population but specifically in men, patients with one or multiple vs. no vascular event showed a different pattern of stroke etiology and of other EVT strategies. In the adjusted analysis, patients overall and men alone with one or multiple vs. no previous vascular events showed more recurrent/progressive in-hospital ischaemic strokes. Patients with multiple vs. no previous vascular events showed higher mortality at discharge and less independency at 3 months. In patients with one or multiple vs. no previous vascular events, men had more in-hospital sICH than women.

### Prevalence of symptomatic vascular disease

Previous studies have examined the *prevalence of co-existing symptomatic vascular disease* in patients with ischaemic stroke but this was prior to the introduction of EVT. In the OXVASC study (a population-based study which focused on patients with minor strokes and TIAs) (3), 27.9% of patients had a history of previous symptomatic vascular disease. In the REACH registry (a prospective study) (5, 6), 40% of patients with cerebrovascular disease had previous symptomatic vascular disease affecting additional vascular beds. In the CRUSADE registry (a study with focus on coronary artery disease) (20), 12.8% of patients had known disease in at least two vascular territories. Our study revealed, that two out of every five patients had one or multiple previous vascular events. The variations in the reported percentages among these studies may be attributed to differences in settings, patient groups, and definitions of co-existing symptomatic vascular disease. Furthermore, our findings suggest that with increasing life expectancy and disease prevalence, there will be a growing number of patients with multiple vascular diseases. This poses a major challenge for healthcare providers in managing these complex patients.

### Association with vascular risk factors

Both, the OXVASC study (3) as well as the CRUSADE registry (20) have demonstrated that the *severity of vascular disease is closely associated with an increased burden of vascular risk factors*.

In the OXVASC study, hypercholesterolemia (OR 6.80, *p* < 0.0001) exhibited a particularly strong association with triple-vascular territory disease. Moreover, arterial hypertension (age/sex-adjusted OR 3.43, *p* < 0.0001), diabetes mellitus (OR 2.89, *p* < 0.0001), hypercholesterolemia (OR 4.67, *p* < 0.0001) and current or previous smoking (OR 1.52, *p* < 0.0001) were more often found in patients with multiple- vs. single-vascular territory disease. In the CRUSADE registry (20), risk factors such as arterial hypertension (*p* < 0.0001), diabetes mellitus (*p* < 0.0001), dyslipidaemia (*p* < 0.0001), renal insufficiency (*p* < 0.0001), and prior congestive heart failure (*p* < 0.0001) were closely related to the number of affected vascular territories.

In our study, we also observed that patients who had experienced one or more vascular events had a greater prevalence of vascular risk factors, such as high blood pressure (*p* < 0.0001), diabetes mellitus (*p* < 0.0001), and abnormal lipid levels (*p* < 0.0001), when compared to patients without any previous vascular events. There was also a trend toward a higher burden of atrial fibrillation, particularly among women with one or multiple vascular diseases (*p* < 0.0001). Atrial fibrillation and multiple vascular diseases often coexist and share common risk factors, including age, high blood pressure, diabetes mellitus, and obesity (21, 22). The presence of multiple vascular diseases can further complicate the management of atrial fibrillation, particularly with regard to antithrombotic therapy. In our study, there is a discrepancy between atrial fibrillation and NOAC/OAC on admission. This difference indicates that in most patients atrial fibrillation was newly discovered on admission.

It is important to note that the studies we and others have conducted primarily focused on classical vascular risk factors, most of which have been found to be more prevalent in men. However, the evaluation of specific vascular risk factors, such as hormone status, factors related to pregnancy, and age at menopause, may introduce new considerations for risk assessment in women (23).

### Preventive medication

In addition, the OXVASC trial (3), the REACH registry (5, 6) and the CRUSADE registry (20) have established that *patients with advanced vascular disease are more likely to use preventive medications* to reduce the risk of vascular events. In the REACH registry (6), it was also noted that the utilization of preventive antiplatelet and lipid-lowering drugs was more prevalent among men compared to women (93.5% vs. 90.1%, *p* < 0.0001 and 73.6% vs. 71.4%, respectively), and this difference could not be entirely explained by variations in vascular risk factor profiles. Our study aligns with these earlier findings. We observed that patients with one or more previous vascular events were more likely to be on preventive medications prior to stroke, even when we analyzed men and women separately.

Interestingly, we also observed lower LDL values in patients with a higher burden of vascular events, indicating treatment adherence. However, patients still failed to meet secondary prevention LDL treatment goals. This underscores the necessity for not only more intensive and consistently adhered treatment, but also for rigorous control of vascular risk factors, including regular follow-up and personalized lifestyle interventions. Conversely, HbA1c levels were significantly higher in patients with a higher burden of vascular events. Additionally, this effect was more pronounced in women than in men, highlighting the urgent need for improved treatment strategies in women.

### Stroke etiology

In the overall study population, and particularly in men, we observed a distinct *pattern of stroke etiology* among patients with multiple previous vascular events in our study. A similar observation was made in the OXVASC study, which also noted a different stroke etiology pattern in patients with multiple-territory vascular disease compared to those with single-territory disease (4). However, in contrast to the OXVASC study, we identified higher rates of cardioembolic strokes in patients with more extensive vascular disease. This divergence in findings is likely due to variations in study settings and patient populations. Interestingly, when we conducted separate analyses by sex, we found that atrial fibrillation was a significant factor contributing to vascular risk in women, but not in men. This observation is consistent with the existing literature (24, 25).

### Therapy modality

In our study, there is a slight difference connected with *applied therapy modality* as patients with one or multiple vs. no previous vascular events had higher rates of receiving mechanical thrombectomy only as opposed to combined treatment strategies. The selection of treatment approach for patients with multiple vascular diseases has not been thoroughly studied in randomized controlled trials and may depend on both the vascular condition and the potential risks of complications. Looking only at men, those without a history of previous vascular events had longer EVT durations and higher rates of extracranial stent placements. This observation may be attributed to the likelihood that these patients had a higher rate of large artery atherosclerosis as the underlying cause of their stroke. Previous research has indicated that EVT procedures may take longer in patients with large artery atherosclerosis due to the challenges of accessing the affected area, fewer first-past successes, and a higher need for permanent stent placements (26, 27).

### Treatment outcomes

The association of *vascular risk profile and treatment outcomes after EVT* was indirectly assessed in previous studies (28–30). In those studies comorbidities such as cerebrovascular and cardiovascular diseases were assessed as part of Hospital Frailty Risk Scores and were shown to be short and long term outcome predictors after EVT.

In our study, in the adjusted analysis, patients overall and men alone with one or multiple vs. no previous vascular events showed more recurrent/progressive in-hospital ischaemic stroke and patients with multiple vs. no previous vascular events experienced more often death at discharge and less independency at 3 months. Poor outcome, higher in-hospital mortality rates, increased risk of stroke recurrence and vascular death in patients with multiple-territory vascular disease were consistently observed across several studies, including the OXVASC study, the REACH registry, and the CRUSADE registry (3, 6, 20). In all of these studies, including our own study, men were more likely to be affected by multiple-territory vascular diseases.

In our analysis we also found that men with one or multiple vs. no previous vascular events were more affected by in-hospital ICHs. To the best of our knowledge, previous studies (12, 31–33) have not established sex as a predictor of symptomatic intracerebral hemorrhage (sICH) following EVT. Possible explanations for this result are the higher prevalence of prior haemorrhagic strokes, pre-stroke antithrombotic medication use, and smoking in our study (34).

Finally, an interesting finding from our multivariable analysis is that women with one or multiple vs. no previous vascular events did not show any difference in their *outcome after EVT*. This result may be attributed to various factors, including comorbidities among women. Additionally, the limited sample size of women with a history of previous vascular disease may have resulted in limited statistical power to detect differences.

### Outlook

It may be necessary to consider sex-specific calculations for the risk of recurrent vascular events and complications, as well as evaluating the need for different follow-up regimens for women compared to men.

Additionally, it has previously been pointed out that high-risk patients could benefit more from stricter preventive measures. A more intensive lipid-lowering approach (3, 35–37) and the inclusion of novel promising therapies such as PCSK9 inhibitors, interfering RNA (siRNA) agents, and inhibitors of angiopoietin-like protein 3 (ANGPTL3) have the potential to improve outcomes (38–41). Various approaches concerning the choice of antiplatelet therapy in high-risk patients have been analyzed in several randomized controlled trials (RCTs) (42–45). More intensive antithrombotic strategies were successfully investigated in the COMPASS study (46). The authors reported a 50% relative reduction in the risk of ischaemic stroke in patients with carotid artery disease and concomitant coronary or peripheral artery disease when using the combination of rivaroxaban plus aspirin compared to aspirin alone. However, it is important to note that the results were limited by an increased risk of major bleeding, predominantly gastrointestinal bleeding.

Future studies should also consider the selection of the therapy approach, as patients with multiple vascular diseases often receive more aggressive preventive treatments that may be incompatible with certain acute therapies. Furthermore, these patients frequently experience complications and unfavorable outcomes following treatment.

### Strengths

The strength of this study is the large cohort of EVT-eligible LVO patients. Furthermore, the study was conducted in an experienced stroke center with an established stroke pathway.

### Limitations

The main limitation of this study is its retrospective analysis and monocentric design. In addition, patients were included over a long period of time during which guidelines, treatment strategies and devices have evolved. We must also acknowledge the potential impact of referral bias, as our institution is a tertiary stroke center providing advanced EVT, which may have resulted in more severe or complex stroke patients being referred to our center. Moreover, the sample size was partly rather small, especially for some outcome variables. Furthermore, we did not assess sex-specific vascular risk factors such as hormone status, pregnancy, or age at menopause. It is also important to note that both biological differences and social aspects related to sex/gender may have influenced our results. Therefore, further research focusing on identifying sex/gender-specific factors could improve the accuracy of risk assessment, refine treatment strategies and address inequalities.

## Conclusions

Previous vascular events increased the risk of in-hospital complications and poorer outcome in the analyzed patients with EVT-eligible LVO-AIS. Our findings may support risk assessment in stroke patients and could contribute to the design of future studies assessing the potential influence of previous symptomatic vascular disease and sex.

## Data availability statement

The original contributions presented in the study are included in the article/Supplementary material, further inquiries can be directed to the corresponding author.

## Ethics statement

The studies involving humans were approved by the local Ethics Committee (KEK Bern 2016–01905). The studies were conducted in accordance with the local legislation and institutional requirements. The participants provided their written informed consent to participate in this study.

## Author contributions

MP: Conceptualization, Data curation, Writing – original draft, Writing – review & editing. GP: Data curation, Formal analysis, Methodology, Writing – original draft, Writing – review & editing. KG: Writing – original draft, Writing – review & editing. MK: Writing – original draft, Writing – review & editing. AB: Writing – original draft, Writing – review & editing. MG: Writing – original draft, Writing – review & editing. MM: Writing – original draft, Writing – review & editing. EW: Writing – original draft, Writing – review & editing. MDM: Writing – original draft, Writing – review & editing. HH: Writing – original draft, Writing – review & editing. PB: Writing – original draft, Writing – review & editing. SB: Writing – review & editing, Writing – original draft. BS: Writing – original draft, Writing – review & editing. AH: Writing – original draft, Writing – review & editing. RU: Writing – original draft, Writing – review & editing. SP-P: Writing – original draft, Writing – review & editing. JG: Investigation, Methodology, Writing – original draft, Writing – review & editing. PM: Investigation, Methodology, Writing – original draft, Writing – review & editing. KA: Data curation, Validation, Writing – original draft, Writing – review & editing. MH: Conceptualization, Data curation, Formal analysis, Investigation, Methodology, Project administration, Supervision, Validation, Writing – original draft, Writing – review & editing.
